# Oat Attenuation of Hyperglycemia-Induced Retinal Oxidative Stress and NF-*κ*B Activation in Streptozotocin-Induced Diabetic Rats

**DOI:** 10.1155/2013/983923

**Published:** 2013-01-10

**Authors:** Abdulrahman L. Al-Malki

**Affiliations:** Department of Biochemistry, Faculty of Science, King Abdulaziz University, P.O. Box 80203, Jeddah 21589, Saudi Arabia

## Abstract

The overproduction of reactive oxygen species (ROS) plays a central role in the pathogenesis of endothelial damage in diabetes. To assess the effect of oat on experimental diabetic retinopathy, five groups of Albino rats were studied: nondiabetic control, untreated diabetic, and diabetic rats treated with 5%, 10%, and 20% (W/W) oat of the diet for 12 weeks. Novel data were obtained in this study indicating a protective role of oat against oxidative stress and diabetic retinopathy. The effects of oat on parameters of oxidative stress, AGE, and nuclear factor kappa B (NF-*κ*B) were assessed by ELISA and NF-*κ*B activation by electrophoretic mobility shift assay. Tumor necrosis factor alpha (TNF*α*) and vascular endothelial growth factor (VEGF) were also determined. After 12 weeks of diabetes, oat treatment reduced blood glucose levels, HbA1c, all oxidative stress markers, CML, normalized NF-*κ*B activation and TNF*α* expression. Furthermore it reduced VEGF in the diabetic retina by 43% (*P* < 0.001). In conclusion, oat modulates microvascular damage through normalized pathways downstream of ROS overproduction and reduction of NF-*κ*B and its controlled genes activation, which may provide additional endothelial protection.

## 1. Introduction

Diabetes is a wide distributed disease characterized by high concentrations of the circulating glucose. It is a lifelong progressive disease and results from body's inability to either produce insulin or use insulin to its full potential. Diabetes is the fourth leading cause of death by disease globally, every 10 seconds a person dies because of the late diabetic complications. Diabetes is a disease which can be controlled but it does not go away. In diabetes, the chronic hyperglycaemia attacks both macrovessels and microvessels throughout the body. In many of the developed countries, diabetes is the leading cause of noninjury amputation, blindness and visual impairment, and end-stage renal disease in adults. It can threaten vision; patients with diabetes develop cataracts at an earlier age. The risk to get glaucoma is nearly twice in diabetic patients compared to nondiabetics [1]. In addition, wound healing impairments is a direct cause of diabetes, and diabetic patients are two to four times more likely to develop cardiovascular disease than people without diabetes.

Diabetic retinopathy, a disease of the retina, is the leading cause of acquired blindness in adults. The microvasculature of the retina is damaged, the blood vessels swell and leak fluid, and, if not prevented, new vessels start to grow and ultimately lead to the detachment of the retina [2, 3]. It is a duration-dependent disease that develops in stages; the incidence of retinopathy is rarely detected in the first few years of diabetes, but the incidence increases to 50% by 10 years, and to 90% by 25 years of diabetes. The prevalence of diabetic retinopathy is increasing due to the prolonged survival of diabetic patients.

Continued high concentrations of the circulating glucose in this life-long disease can damage retina via many acute (and/or repeated) and also cumulative long-term changes. Although the capillaries of the retina are lined with endothelial cells that maintain the blood retinal barrier and are supported with an equal number of pericytes that provide tone to the vessels, the ratio of endothelial cells to pericytes in diabetes is altered to be about 4 : 1 [4]. All the blood vessels of the retina have tight junctions that help to protect them against leaking, but prolonged high concentrations of glucose damage these tight junctions and the vessels become leaky allowing the fluid or/and blood to seep into the retina, which results in the swelling of the retina [5]. Due to progressive dysfunction, the capillaries die prematurely resulting in ischemia that can be followed by neovascularization and finally retinal detachment and blindness [6, 7].

In the development of diabetic retinopathy, the basement membrane thickens and the blood pressure is altered. In addition pericytes and endothelial cells undergo accelerated apoptosis leading to pericyte ghosts and acellular capillaries [8]. The leukocytes become less deformable, and retinal leukostasis is increased affecting the endothelial function [9, 10]. 

The linkage between the development and progression of diabetic retinopathy and any pathway is still largely speculative. Several studies suggest that glucose-induced production of reactive oxygen species (ROS) stimulates several of the biochemical mechanisms that can be involved in hyperglycaemia-mediated complications of diabetes, including retinopathy. Cumulative studies suggest that many related hyperglycaemia-affected pathways play a major role in the pathogenesis of diabetic retinopathy. The most actively studied pathways are the oxidative stress pathway [11], polyol pathway activity [12, 13], formation of advanced glycation end product (AGEs) [14, 15], activation of protein kinase C (PKC) isoforms [16, 17], and increase in augmentation of the hexosamine pathway flux [18]. The linkage between the development of diabetic retinopathy and any pathway is still largely speculative. Previous studies used STZ to induce diabetes in experimental animals [6, 8, 14, 15, 18].

 Cumulative studies demonstrated that dietary fiber can significantly reduce the risk of cardiovascular disease and type 2 diabetes mellitus [19]. This is due in part to the ability of fiber to reduce postprandial glycemia and improve long-term glycemic control [20, 21]. It was postulated that the rheological properties of soluble dietary fibers are highly related to their effects on control of the glucose concentration [22]. For instance, the ability of oat-derived *β*-glucan to reduce postprandial glycaemia has been strongly correlated with its viscosity [23], demonstrating an inverse linear relationship between the logarithm of viscosity measures and peak postprandial plasma glucose and insulin responses after consuming various doses of purified oat *β*-glucan with a 50 g oral glucose load. Despite these findings, the levels of viscosity required to achieve specific glucose-lowering effects are poorly understood. Still, the majority of trials investigating dietary fiber have not accounted for the principles of polysaccharide solubility and viscosity as the main determinants of its physiological outcome. While a small number of studies have shown the effect of oat on diabetes [24, 25], none examine its effect on the development and progression of diabetic retinopathy.

The aim of this study is to evaluate the effect of oat on the hyperglycemia-induced oxidative stress and if this can attenuate the development of diabetic retinopathy. Because oat is natural and widely used, the results of this study may provide an alternative for enhancing nutrition and diabetic control during diabetic retinopathy.

## 2. Materials and Methods

### 2.1. Induction of Diabetic Retinopathy Model and Study Design

Nine-week-old 200 ± 20 g male Albino rats were housed in cages and received normal rat chow diet and tap water ad libitum in a constant environment (room temperature 28 ± 2°C, room humidity 60 ± 5%) with a 12 h light, 12 h dark cycle. The animals were kept under observation for one week prior to the start of the experiments. All procedures were done according to the Animal Ethics Committee. 10 rats were randomly selected as control group (group 1, *n* = 10), which received a single tail vein injection of 0.1 mol/L citrate buffer only. The other 45 rats received a single dose of STZ (Sigma S-0130) in citrate buffer pH 4.5 through the indwelling catheters over 2 min, at a fixed dose of 60 mg/kg [16]. Only rats with blood glucose higher than 250 mg/dL after two days were considered as being diabetic in the fasting state. Blood glucose was measured by using *one touch select* Sensor Analyzer (Life Science, UK). Rats with blood glucose levels lower than 200 mg/dL were excluded from the study. All studies were carried out two days after STZ injection. Diabetic rats were classified to four groups each contains ten rats: group 2, untreated diabetic untreated rats (*n* = 10) and groups 3–5 (*n* = 10 rats each), oat treated diabetic rats. Rats of these groups were supplemented with oat 5, 10, and 20%, respectively, on the diet (W/W). Treatment was continued for 12 weeks starting from day two after STZ administration. At the end of the experiments, the final body weight of the various groups was recorded. Then, animals were fasted overnight (18 h) and then anesthetized [26]. Blood was collected directly from the heart of each animal. Serum was used for the determination of glucose, total protein, and albumin using a Cobas integra 800 automatic analyzer of Roche Diagnostic (USA) according to the instructions of the manufacturer. Rat eyes from each group were removed, washed with cold normal saline, and used for preparation of the eye homogenate and histopathological examinations.

### 2.2. Eye Homogenate Preparation

Retinal protein was extracted from freshly enucleated eyes (*n* = 9 from diabetic groups and *n* = 6 from the control group) and processed as described [26]. The isolated individual retinae were rinsed thoroughly with ice-cold phosphate-buffered saline to remove blood components and homogenized in a lysis buffer (containing 63 mmol/L Tris-HCl, pH 6.8; 1% Nonidet P-40; 0.25% SDS; 150 mmol/L NaCl; 1 mmoL/L EDTA; 5 mmol/L EGTA; 1 mmol/L phenyl methyl sulphonyl fluoride; 1 *μ*g/mL of aprotinin and leupeptin; 2 mmol/L benzamidine; 1 mmol/L NaF; 10 nM okadaic acid; and 0.1% SDS). The supernatant was aliquoted and stored at −80°C and assayed for protein concentration using BCA kit (Pierce, Rockford, USA) using albumin diluted in lysis buffer as standard. 

Part of the retina was homogenized with 100 *μ*L TOTEX buffer (100 mM HEPES-KOH, pH 7.9, 0.35 M NaCl, 20% glycerol, 1% NP-40, 1 mM MgCl_2_, 0.5 mM EDTA, 0.5 mM EGTA, 10 *μ*g/mL leupeptin, 0.5 mM DTT, 0.2 mM PMSF) for 30 seconds, incubated in ice bath for 30 minutes, vortexed, and centrifuged at 13000 rpm for 5 minutes. The supernatant which contained the total retinal extract was transferred to a fresh tube and kept at −80°C for electrophoretic mobility shift assay (EMSA) [27].

### 2.3. Determination of Reduced GSH

Glutathione (GSH) plays a central role in protecting mammalian cells against damage incurred by free radicals, oxidants, and electrophiles. Reduced GSH was measured by colorimetric end-point assay using dithionitrobenzoic acid method as described by Moron et al. and Mekheimer et al. [28, 29]. GSH concentration was expressed as *μ*mol/mg protein using GSH Kit from Roche (Mannheim, Germany) according to the instructions of the manufacturer.

### 2.4. Determination of Lipid Peroxidation

The concentration of TBARS was determined as MDA according to Okhawa et al. and Sayed [30, 31]. The degree of lipid peroxidation in the retina was determined using the Bioxytech LPO-586 kit. The concentration of MDA was expressed in terms of nmol/mg protein.

### 2.5. Determination of SOD Activity

SOD activity was determined as the volume of homogenate that is required to scavenge 50% of the superoxide anion generated from the photo illumination of riboflavin in the presence of EDTA (1 unit of SOD activity) [32, 33]. The activity was determined using the SOD available kit from BioVision Research Products (Linda Vista Avenue, USA) according to the instructions of the manufacturer.

### 2.6. Assessment of Retinal CML

The supernatant of the retinal homogenate was tested for CML using the anti-CML rat autoantibody ELISA kit which employs the semiquantitative enzyme immunoassay technique. The absorbance of the resulting yellow product is measured at 450 nm [34–36]. The levels of CML of the retinal extract were determined using the ELISA kit from Roche Diagnostics (Mannheim, Germany) according to the instructions of the manufacturer.

### 2.7. Assay of TNF*α*  and VEGF

The levels of TNF*α*  and VEGF in the isolated retinal proteins were determined as previously described [37], using a specific rat ELISA kit. The ELISA kits were obtained from BD Biosciences, Pharmingen (San Diego, CA, USA). Determination of TNF*α*  and VEGF were performed according to the manufacturer's instructions. The reaction was stopped and absorbance was read immediately on an ELISA reader (Model 3550, BIO-RAD Laboratories, Ca, USA). The levels of TNF*α*  and VEGF were expressed as pg/mg protein.

### 2.8. Electrophoretic Mobility Shift Assay (EMSA)

The retinal extract was assayed for the transcription factor binding activity using the NF-*κ*B-p65 consensus sequence: 5′-AGTTGAGGGGACTTTCCCAGGC-3′. Specificity of binding was ascertained by competition with a 160-fold molar excess of unlabeled consensus oligonucleotides as previously described [38, 39]. EMSA experiments were performed at least three times.

### 2.9. Histopathological Examinations

After 12 weeks of the experiment, rats were scarified and eyes were enucleated and fixed in formalin. Sections of the entire globe were prepared, stained with haematoxylin and eosin (H&E), and examined by light microscope.

### 2.10. Statistical Analysis

Statistical analysis was performed using the SPSS software. The effect of each parameter was assessed using the one way analysis of variance. Individual differences between groups were examined using Dunnett's test and those at *P* < 0.05 were considered statistically significant*.*

## 3. Results

### 3.1. Blood Biochemical and Physiological Parameters

The weights of the rats at the beginning of the study were similar in all groups. At the end of the experiment, diabetic animals presented a significant weight loss. The initial and final body weights were not significantly different in groups 1, 3, 4, and 5 (Table 1). Treatment of rats with STZ resulted in a significant increase in blood glucose levels in the diabetic untreated group compared with the control group (*P* = 0.01), while treatment with 5, 10, and 20% oat resulted in a significant decrease in blood glucose compared with the untreated diabetic animals (*P* < 0.05, Table 1). As a result of diabetes, HbA1c and total protein were significantly increased (*P* < 0.05) in the diabetic untreated group. Treatment of animals with oat improves the two parameters in a dose-dependent manner (Table 1). The concentration of serum albumin was not affected by STZ treatment. 

### 3.2. Oxidant/Antioxidant Parameters

The antioxidant enzyme activities like catalase, glutathione reductase, and glutathione peroxidase and the concentration of reduced GSH in the blood of diabetic animals were significantly reduced as a result of STZ treatment. Supplementation of oat resulted in a significant increase of the activity of these enzymes and in the level of GSH (Table 1). This increase in the activity of the antioxidant enzymes was found to be dose-dependent. In addition, the oxidative stress biomarkers in the retina were assayed. The activities of catalase, SOD, glutathione-S-transferase, and glutathione peroxidase were significantly reduced as a result of STZ administration. As a result of oat supplementation, the reduced activities of the antioxidant enzymes were increased in a dose-dependent manner (Table 2). The degree of lipid peroxidation in the retina was significantly elevated as a result of diabetes. Administration of oat resulted in a dose-dependent decrease of MDA levels (Table 2). 

### 3.3. Effects of Oat on the Retinal CML

As a result of diabetes the production of AGEs increases. In the present study we measure the retinal levels of CML. CML was significantly elevated in the STZ-diabetic rats compared with the normal control rats. Treatment of rats with 5, 10, and 20% (W/W) oat in the diet for 12 weeks resulted in lowering of these elevated levels. The reduction of CML formation was found to be significant and dose-dependent when compared with the diabetic untreated rats (Table 2). 

### 3.4. Effect on the Levels of TNF*α*  and VEGF

**Figure 1:**
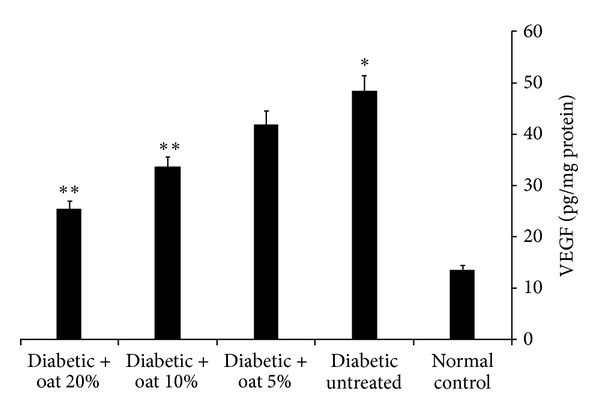
Retinal VEGF levels assessed at 12 weeks of oat treatment. VEGF was significantly higher in the diabetic untreated group than in the normal control group. Oat treatment resulted in a dose-dependent reduction of the elevated VEGF levels. Data are mean ± SE (*n* = 10 retinas/group), *P < 0.01 versus normal control, and **P < 0.01 versus diabetic untreated group.

**Figure 2:**
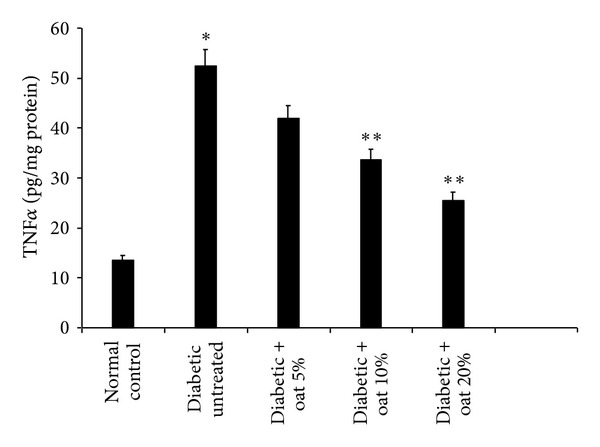
Retinal TNF*α*  levels assessed at 12 weeks of oat treatment. TNF*α*  was markedly elevated in the diabetic untreated group than in the normal control group. Tretment of rats with oat resulted in a dose-dependent reduction of the elevated TNF*α*  levels. Data are mean ± SE (*n* = 10 retinas/group), *P < 0.01 versus normal control, and **P < 0.03 versus diabetic untreated group.

Both TNF*α*  and VEGF play an important role in the pathogenesis of diabetic retinopathy. The levels of TNF*α*  and VEGF were measured in the retina of the rats. As a result of STZ injection the retinal levels of TNF*α*  and VEGF were significantly elevated indicating a considerable level of inflammation compared with the normal control rats. Administration of 5, 10 and 20% (W/W) oat in the diet for 12 weeks resulted in a significant and a dose-dependent reduction of these elevated levels. This reduction was shown in Figures 1 and 2.

### 3.5. Histopathological Findings

**Figure 3:**
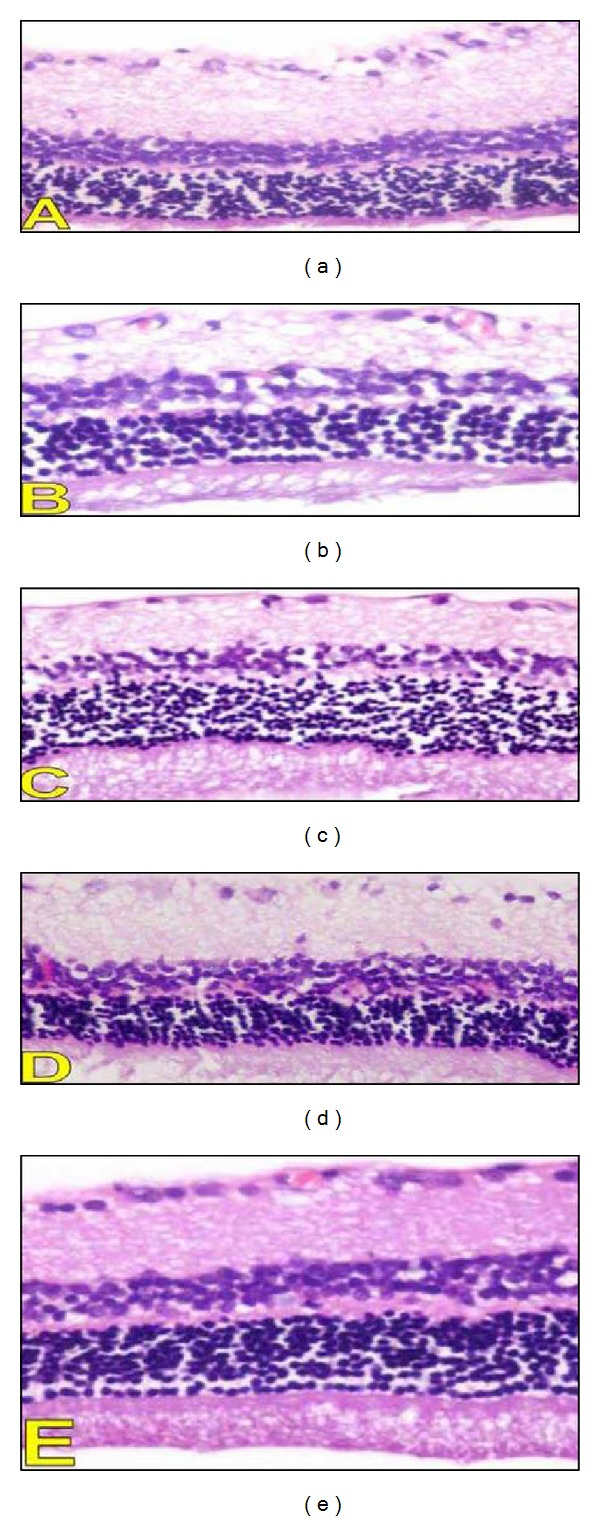
Histopathological findings in the retina. A photomicrography representing H&E sections from different studies groups (A: diabetic group, B–D: diabetic rats treated with 5, 10 and 20% oat in the diet (W/W) respectively, and E: Control group) (200x).

On microscopic examination, the layers of retinae were intact in all animal groups (Figure 3). There was no significant difference in the cellular content of the ganglion cell layer. A previous study showed that the observation of retinopathy in normal H&E staining can be seen only 30 weeks after induction of diabetes [40, 41]. 

### 3.6. Effects of Oat on the Activation of NF-*κ*B

**Figure 4:**
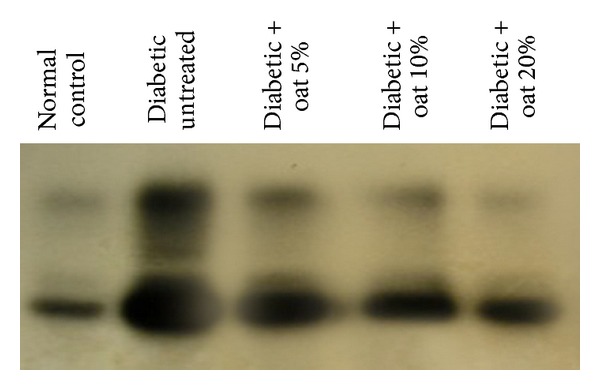
EMSA for NF-*κ*B for total retinal extract. Compared with the normal group, the diabetic untreated group shows markedly increased NF-*κ*B expression and the oat-treated groups show a dose-dependent decreased expression.

As a result of diabetes and enhanced formation of AGEs, NF-*κ*B-p65 was markedly activated in the diabetic untreated group compared with the normal control group. The activation of NF-*κ*B-p65 was due to the high concentration of AGEs. These elevated AGEs interact with their receptor RAGE resulting in the obtained activation of NF-*κ*B-p65. Oat supplement resulted in a significant reduction of the activated NF-*κ*B-p65 in a dose-dependent manner as indicated in Figure 4. As mentioned in Table 2, oat attenuated AGE (in particular CML) production which resulted in the reduction of NF-*κ*B-p65 in Figure 4.

## 4. Discussion

The present study demonstrated that the interference with the overproduction of ROS by oat in the diabetic rats normalizes parameters of oxidative stress in the diabetic retina and prevents the activation of major pathways involved in hyperglycaemia-induced vascular damage. Oat reduced or even normalized downstream effectors of vascular response to injury. These *in vivo* data show an evidence of oxidative protein modification. Earlier short-term studies using antioxidants showed inhibition of retinal lipid peroxidation products and superoxide dismutase, but no change in enzymes of glutathione metabolism [42]. In addition, the reduction of ROS overproduction and the associated reduction of intracellular CML suggest an indirect AGE-inhibiting effect of oat. 

RAGE is an important receptor for mediating AGE effects [43]. RAGE is normally expressed in the inner vascularised part of the diabetic retina, both in neuroglial and in vascular cells [44]. Binding of AGE such as CML to RAGE enhances oxidative stress in retinal tissue [44]. RAGE signals via NF-*κ*B activate target genes which have a harmful potential for the diabetic retina [45]. Oat reduced NF-*κ*B-p65 antigen and NF-*κ*B binding activity. Thus, our data suggest that part of the beneficial effects of oat includes the disruption of the detrimental AGE-RAGE-NF-*κ*B pathways.

Both preclinical and clinical studies have shown the significance of VEGF in the pathogenesis of proliferative diabetic retinopathy [46, 47]. Similarly, in the present study expression of retinal VEGF has been found to be significantly increased in diabetic untreated rats compared to the normal control rats, while oat treatment attenuated the expression of VEGF. Various studies have shown that antioxidants inhibit VEGF-mediated angiogenesis [48]. Eventually, antioxidants have been found to inhibit angiogenesis by abrogating VEGF signaling through interfering with the formation of VEGF receptor 2 complex which may have a physiological significance in the management of diabetic retinopathy [49]. 

TNF-*α*  contributes to the pathogenesis of diabetic retinopathy [50], and significantly higher levels of TNF-*α*  are found in the plasma of patients affected by either type 1 or type 2 diabetes versus age-matched healthy control subjects [51]. Earlier, the role of inflammatory cytokine TNF-*α*  in the apoptotic cell death of retinal endothelial cells during early and late stages of diabetic retinopathy in a rat model of streptozotocin-induced diabetes has been investigated [52]. Similarly, in the present study TNF-*α*  expression was significantly increased in the diabetic untreated group compared with the normal control group, and the oat-treated groups showed significantly lower levels of TNF-*α*  than the diabetic untreated group. Other studies also reported that antioxidants dose-dependently inhibit inflammatory markers like TNF-*α*, IL-6, and IL1*β* gene expression in the cells of chronic diseases and their release from a human cancer cell line [53–58]. 

In summary, the present study clearly demonstrates that both of controlling of hyperglycaemia and catalytic scavenging of reactive oxygen species are effective approaches for the prevention of diabetic retinopathy. Oat is a paradigm natural food supplement with a broad spectrum of beneficial biochemical and cell biological effects, based on its ability to reduce the hyperglycaemia-induced ROS overproduction. Since oat also has beneficial effects on other target tissues of diabetic angiopathy and shows beneficial effects of mediators of large vessel damage, this concept appears attractive for the prevention or delay of diabetic angiopathy. In conclusion, it can be postulated that oat could have potential benefits in the prevention of the onset and progression of retinopathy in diabetic patients.

## Figures and Tables

**Table 1:** Initial and final body weights, blood biochemical, and physiological parameters of the rats.

	Group 1	Group 2	Group 3	Group 4	Group 5
Initial body weight, g	195.13 ± 9.5	196.44 ± 7	194.19 ± 6	197 ± 7.2	196.33 ± 6.5
Final body weight, g	265.5 ± 8.2	161.8 ± 7.2^a^	203.13 ± 16.5^a,b^	215.52 ± 8.9^a,b^	234 ± 12.3^a,b^
Glucose, mg/dL	92.78 ± 0.45	265.35 ± 1.45^a^	115.92 ± 1.4^a,b^	106.71 ± 1.23^b^	96.21 ± 0.57^a,b^
HbA1c, %	5.24 ± 0.41	9.42 ± 0.34^a^	7.88 ± 0.54^ b^	6.85 ± 0.38^ b^	6.88 ± 0.52^ b^
Albumin, g/L	38.4 ± 5.7	38.2 ± 7.7	38.5 ± 6.5	38.7 ± 7.5	38.9 ± 8.1
Total protein, g/L	76 ± 7	62 ± 6.5^a^	63.2 ± 6.5^a^	65.6 ± 7.1	69.91 ± 7.5^b^
GSH, mmol/L	0.281 ± 0.015	0.105 ± 0.03^a^	0.116 ± 0.02^a^	0.145 ± 0.021^a,b^	0.172 ± 0.02^a,b^
Catalase, U/gHb	94.54 ± 14	50.98 ± 15^a^	62.13 ± 13^a,b^	70.11 ± 11^a,b^	86.55 ± 12^b^
Glutathione reductase, U/gHb	4.25 ± 0.09	2.25 ± 0.62^a^	2.31 ± 0.71^a,b^	2.54 ± 0.54^a,b^	3.21 ± 0.94^b^
Glutathione peroxidase, U/gHb	57.1 ± 11	145.5 ± 45^a^	129 ± 11.1^a,b^	89.54 ± 10.2^b^	69.9 ± 9.55^b^

Data are expressed as the means ± SD. Group  1: normal control; group 2: diabetic untreated and groups 3–5: diabetic rats treated with 5, 10, and 20% (W/W) oat in the diet, respectively. Each group consisted of 10 animals.

^a^ *P* < 0.05 versus normal control group, ^b^ *P* < 0.05 versus diabetic untreated group.

**Table 2:** Oxidant/antioxidant parameters as well as concentration of CML in the rat retina.

	Group 1	Group 2	Group 3	Group 4	Group 5
MDA, nmol/mg protein	2.45 ± 0.16	4.75 ± 0.17^a^	3.98 ± 0.52^a,b^	3.15 ± 0.25^ a^	2.95 ± 0.15^b^
GST, nmol substrat·mg protein^−1^·min^−1^	176 ± 31	82 ± 13^a^	105 ± 17^a,b^	125 ± 11^a,b^	151 ± 9^a,b^
GSH-Px, nmol substrat·mg protein^−1^·min^−1^	0.92 ± 0.17	0.34 ± 0.09^a^	0.55 ± 0.1^a,b^	0.67 ± 0.15^ a,b^	0.82 ± 0.165^b^
Catalase, IU·mg protein^−1^	2.61 ± 0.032	0.52 ± 0.03^a^	0.56 ± 0.02^a^	0.96 ± 0.04^a,b^	1.45 ± 0.21^a,b^
SOD, nmol substrat·mg protein^−1^·min^−1^	3.53 ± 0.45	1.82 ± 0.35^a^	1.92 ± 0.5^a^	2.3 ± 0.6^a^	2.95 ± 0.75^b^
GSH, nmol/mg protein	16 ± 3	16.11 ± 2	16.1 ± 2.1	15.95 ± 2	16 ± 2.3
CML, pg/mg protein	3.54 ± 0.22	8.81 ± 0.34^a^	5.42 ± 0.35^a,b^	3.85 ± 0.55^b^	3.2 ± 0.6^b^

Data are expressed as the means ± SD. Group  1: normal control; group 2: diabetic untreated group; groups 3–5: diabetic groups treated with 5, 10, and 20% (W/W) oat in the diet, respectively. Each group consisted of 10 animals.

^a^ *P* < 0.05 versus normal control group, ^b^ *P* < 0.05 versus diabetic untreated group.

## Abbreviations

CML: Carboxymethyllysine
MDA: Malondialdehyde
TBARS: Thiobarbituric acid reactive substance
SOD: Superoxide dismutase
HEPES: 4-(2-Hydroxyethyl)-1-piperazineethanesulfonic acid
NP-40: Nonylphenoxy polyethoxy ethanol
EGTA: Ethylene glycol-bis(*β*-aminoethyl ether)
DTT: Dl-dithiothreitol
PMSF: Phenylmethylsulfonyl fluoride.
